# Transient but recurrent complete heart block in a patient after COVID-19 vaccination – A case report

**DOI:** 10.1016/j.amsu.2022.103694

**Published:** 2022-04-29

**Authors:** Hoffer Etienne, Pirlet Charles, Troisfontaines Pierre

**Affiliations:** Department of Cardiology, CHR Citadelle Hospital, Liège, Belgium

**Keywords:** Case report, COVID-19, Atrioventricular block, Vaccine

## Abstract

**Introduction:**

Cardiovascular disorders have been extensively reported during COVID-19 illness, including arrhythmias such as atrioventricular conduction disturbances. To date, one case of transient heart block has been reported after COVID-19 vaccine.

**Case presentation:**

A 73 years-old woman presented with shortness of breath and fatigue 2 weeks after receiving her second dose of BNT162b2 SARS-CoV-2 mRNA vaccine. ECG showed complete AV block with normal narrow QRS complexes. Chronic treatment with Bisoprolol for hypertension was stopped but complete AV block persisted 48 hours thereafter. Therefore, a permanent pacemaker was implanted. Three months later, pacemaker follow-up revealed no ventricular stimulation, suggesting complete recovery of AV conduction, even after resumption of bisoprolol. Five months after the second dose, she received a third dose of the same vaccine. Three weeks later, she once again complained of dyspnea on exertion. ECG showed sinus rhythm with permanent ventricular stimulation. After device inhibition, complete AV block was confirmed and, 2 weeks later, conduction was restored once more.

**Clinical discussion:**

It is known that vaccines can induce AV conduction disturbances, mostly reversible. The underlying mechanisms leading to high-degree AV block remain unclear and are probably multiple. Although being exceptional after COVID-19 vaccine, our case illustrates the fact that the latter can induce such a disturbance which may be transient and recurrent even in the absence of underlying conduction disorder.

**Conclusion:**

COVID-19 vaccination may transitorily interfere with cardiac conduction system even in subjects without known underlying heart disease.

## Highlights

• Transient but recurrent complete AV block after COVID-19 vaccine.

## 1 Introduction

Transient or less often persistent high-degree atrioventricular block has been described in COVID-19 hospitalized patients [1,2], particularly when they present with viral myocarditis [3]. One case of transient heart block has been reported in an 80 years-old man after his first shot of COVID-19 BBIBP-CorV (Sinopharm) [4]. We describe a case of a 73 years-old woman who presented twice with complete atrioventricular (AV) block 2 weeks after the second and third shots of SARS-CoV-2 mRNA vaccine. This case report has been reported in line with the CARE Criteria.

## 2 Case presentation

**Fig. 1:**
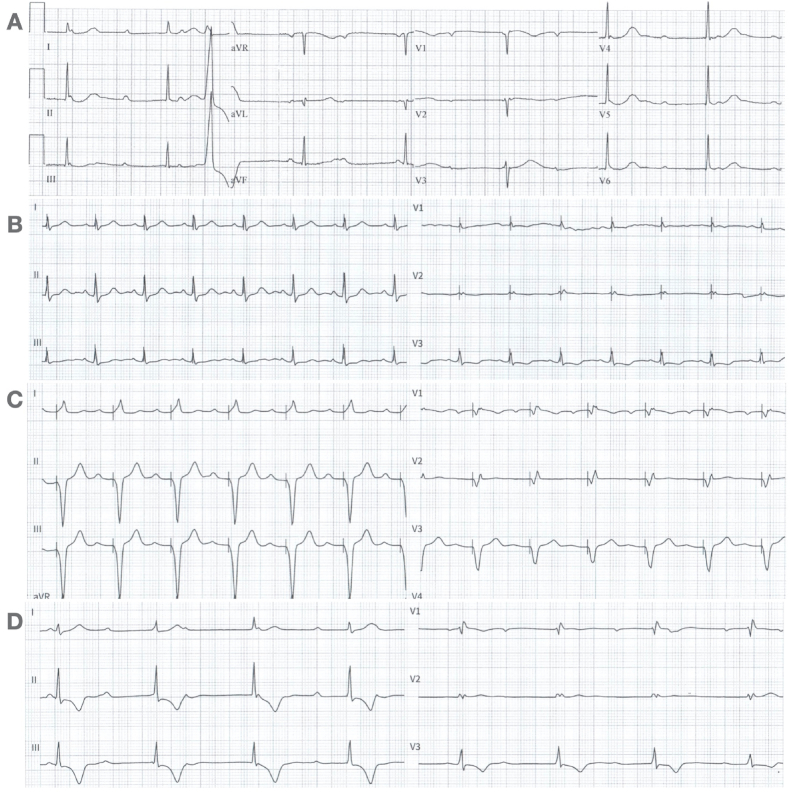
Electrocardiogram on arrival showing complete atrioventricular (AV) block (A). Complete AV conduction recovery three months later (B). After the third dose of the vaccine, a permanent ventricular stimulation was observed (C) due to recurrence of complete AV block (D).

A 73 years-old woman presented with shortness of breath and fatigue 2 weeks after receiving her second dose of SARS-CoV-2 mRNA vaccine (BNT162b2). On arrival, her heart rate was 45 beats per minute (BPM) and blood pressure was 106/67 mmHg. Jugular veins were not congestive. Oxygen saturation was 94% on room air. Cardiopulmonary examination was unremarkable except for the fact that the intensity of the first heart sound decreased when PR intervals were prolonged. Her long-standing medication included bisoprolol 2.5 mg/day taken for sinus tachycardia and systemic hypertension. Electrocardiogram on arrival showed complete AV block with junctional rhythm at around 45 BPM (Fig. 1A). Due to persistence of complete AV block 48 hours after bisoprolol was stopped, it was decided to implant a permanent dual chamber pacemaker. Three months later, the pacemaker follow-up showed no ventricular stimulation, suggesting complete AV conduction recovery, even after bisoprolol was restarted (Fig. 1B). Five months after the second dose, she received a third dose of the same vaccine. Three weeks later, she once again complained of dyspnea on exertion. ECG showed sinus rhythm with permanent ventricular stimulation (Fig. 1C). After device inhibition, complete AV block was observed (Fig. 1D). Four weeks later, AV conduction was restored once again (same as Fig. 1B).

## 3 Discussion

SARS-CoV-2 disease can induce severe respiratory failure. However, it rapidly became apparent that other organs such as the heart could also be affected. Among cardiac manifestations, arrhythmias were commonly reported [5], particularly sinus bradycardia. Bradyarrhythmia may be the clinical manifestation of high-degree AV block. Dagher et al. reported 4 cases of transient complete AV block with a maximal duration of 6 hours [1]. On the other hand, when the phenomenon persists several days, the chance of recovery is low and permanent pacemaker implantation is often indicated [2,3]. The underlying mechanisms leading to high-degree AV block remain unclear and are probably multiple. It has been postulated that high systemic inflammatory burden as well as the cytokine storm could induce myocardial ischemic injury affecting the intrinsic conduction system [6]. Another potential mechanism is a direct viral injury to the myocardium in case of viral myocarditis [7]. In that case, cardiac magnetic resonance imaging may be of interest to detect early cardiac involvement. A third mechanism compromising normal conduction is the overexpression of the angiotensin-converting enzyme 2 receptor (ACE-2) due to SARS-CoV-2. It should be borne in mind that some patients have pre-existing conduction disorders and others are receiving medications that could influence AV conduction.

Induction of high-degree AV block has not been reported in phase I and II COVID-19 vaccine trials. Possible explanations should be that in these early phases, the populations were often limited and mainly included young people, below 60 years. When older people were concerned, ECG abnormalities were very often exclusion criteria. Moreover, phase I and II trials are not designed or powered to detect very rare side effects. Rare cases of vaccine-associated AV block have been reported after small-pox vaccination [8]. The unique case of transient AV block possibly related to COVID-19 vaccine in an 80 years-old man with underlying coronary artery disease and previous left bundle branch block has been reported [4]. The patient developed 2:1 AV block 5 days after his first shot of COVID-19 BBIBP-CorV (Sinopharm). Three days later, the conduction disorder regressed to simple prolonged AV conduction. Due to subsequent intermittent 2:1 block, permanent pacing was decided. Based on their experience, these authors also proposed an algorithm to identify healthy COVID-19 vaccine recipients who are supposed to be at an increased risk of developing worsening AV block.

Nevertheless, the relationship between vaccination and the conduction disorder seems to be more robust in our case:

· Our patient had no underlying cardiac disease and no previous ECG abnormalities
· Complete AV block occurred twice after inoculation of the vaccine, with similar delay
· Normal AV conduction was restored shortly after vaccination

It would of course be of interest to know if future inoculations of COVID-19 vaccines will induce the same phenomenon.

## 4 Conclusion

Although very rare, BNT162b2 COVID-19 vaccine may induce high-level AV block, even in people without underlying conduction system disturbance. In case of bradycardia, an ECG should therefore be performed to exclude such conduction disorders.

Even if reversible, high-level AV block can reappear after a subsequent injection of the vaccine. The decision to implant a permanent pacemaker should therefore be considered on a case-by-case basis.

## Ethical approval

Written informed consent was obtained from the patient.

## Please state any sources of funding for your research

None.

## Author contribution

Hoffer Etienne: Manuscript writing, literature search and final drafting. Pirlet Charles: Literature search and drafting. Troisfontaines Pierre: Supervision from conceptualization to editing final manuscript.

## Consent

Written informed consent was obtained from the patient for publication of this case report and accompanying images. A copy of the written consent is available for review by the Editor-in-Chief of this journal on request.

## Registration of research studies

Name of the registry:
Unique Identifying number or registration ID:
Hyperlink to your specific registration (must be publicly accessible and will be checked):

## Guarantor

Hoffer Etienne, MD.

## Declaration of competing interest

None of the authors has any conflict of interests to declare.
